# A new blind species of the cave genus *Oreonectes* from Guizhou, China (Nemacheilinae)

**DOI:** 10.3897/zookeys.637.10202

**Published:** 2016-11-28

**Authors:** Huaiqing Deng, Huamei Wen, Ning Xiao, Jiang Zhou

**Affiliations:** 1 School of Life Sciences, Guizhou Normal University, Guiyang, Guizhou, 550001, China; 2 Guiyang Nursing Vocational College, Guiyang, Guizhou, 550003, China

**Keywords:** Cavefish, Libo, new species, Oreonectes daqikongensis sp. n.

## Abstract

This study aimed to describe a new specimen of cavefish collected from a karst cave in the Daqikong area of Libo County, Guizhou. Twenty-six cavefish specimens were collected and identified as a new species of Balitoridae: Nemacheilinae, and named *Oreonectes daqikongensis* **sp. n.** A genetic analysis was performed and showed that its genetic distances from *Oreonectes shuilongensis* and *Oreonectes platycephalus* are higher than intraspecific distances. Discovery of this species will be helpful to understand the distribution of *Oreonectes*.

## Introduction

Nemacheilinae are common in tropical Asia. They occur in a great variety of habitats, particularly abundant in swiftly flowing hillside streams. The similar living environment may help explain why many cavefish of Asia belong to this subfamily (Kottelat 1990).

There are 15 genera and more than 100 species was found belong to the Nemacheilinae subfamily in China so far (Zhu 1995). The *Oreonectes* was first established by Günther (1868) with *Oreonectes platycephalus* as the type species. A total of 16 species of Oreonectes are considered valid. Du et al (2008) divide Oreonectes into two groups that including furcocaudalis group and platycephalmus group. The platycephalmus group includes the *Oreonectes platycephalus*, *Oreonectes Polystigmus*, *Oreonectes guananensis* (Yang et al. 2011), *Oreonectes luochengensis* (Yang et al. 2011), and *Oreonectes anophthalmus* (Zhu 1989). The furcocaudalis group includes the *Oreonectes microphthalmus* (Du et al. 2008), *Oreonectes macrolepis*, *Oreonectes retrodorsalis* (Lan et al. 1995), *Oreonectes acridorsalis*, *Oreonectes barbatus*, *Oreonectes duanensis*, and *Oreonectes donglanensis* (Lan et al. 2013), *Oreonectes elongates* (Tang et al. 2012), *Oreonectes translucens* (Zhang et al. 2006), *Oreonectes furcocaudalis* (Zhu 1989), and *Oreonectes* sp. n. (Chen et al. 2011). They all dwell in underground rivers of the karst environment (Yang et al. 2011; Lan et al. 2013). During a cave biodiversity survey on Libo County, Guizhou in 2011, we using seines nets and the bait collected 26 new cavefish specimens at Daqikong area. This study aimed to describe and identify the new specimen of cavefish.

## Materials and methods

The holotype was fixed and preserved in 10% formalin, and the paratypes were preserved in anhydrous ethanol. The specimens were stored in the Animal Specimen Room of the School of Life Sciences, Guizhou Normal University
(GNUG). All measurements are taken on the left side of the fish specimens. All measurements were taken point to point with digital calipers to 0.1 mm. The new species was identified according to the morphological features, molecular phylogenetic evidence, and distribution regions. Counts and proportional measurements follow Tang et al. (2012). The sources of material of other *Oreonectes* species is in Appendix 1.

The tissue sample was extracted from the right side of the specimen no. 25, from which the genomic DNA was extracted using from muscle tissues by standard phenolchloroform methods (Sambrook and Russell 2001). Then the cytb gene segment was amplified using the Cyprinidae universal primers L14724 and H15915 (Rui et al. 2012). Both the amplification and sequencing were completed in Beijing Ruijie Gene Technology Co., Ltd. (Beijing, China).

The Sequence Alignment Editor (BioEdit) software was used to analyze sequencing peaks and delete carrier sequences, and then Seqman v5.51 (DNAStar) was used to perform the sequence assemble and alignment. Complete cytb sequences of other 29 species of Nemacheilinae were obtained from GenBank (Table 1). The cytb gene nucleic acid sequences of all the 30 species were compared with the ClustalW method of MEGA 6.0 software (Tamura et al. 2013), the terminal irregular regions were removed manually. Subsequently, the phylogenetic tree was established using the maximum likelihood (ML) method in MEGA 6.0, while reliability was tested using the Kimma2-Pamameter distance model and bootstrap method by repeating 1000 times.

**Table 1.** GenBank accession numbers for the analyzed samples included in the phylogenetic analysis.

Genus	Species	Accession
*Oreonectes*	*shuilongensis*	KF640641
	*daqikongensis*	KU987436
	*platycephalus*	DQ105197
*Schistura*	*fasciolata*	HM010565
	*caudofurca*	JN837651
	*desmotes*	GQ174368
	*callichroma*	JN837652
	*latifasciata*	JN837653
	*bucculenta*	JN837654
	*macrotaenia*	JN837655
	*amplizona*	JN837656
	*cryptofasciata*	JF340401
	*sikmaiensis*	JF340405
	*poculi*	JF340407
	*longa*	JF340408
*Homatula*	*pycnolepis*	KF041000
	*acuticephala*	HM010527
	*longidorsalis*	HM010550
	*potanini*	JF340388
*Traccatichthys*	*pulcher*	JF340402
*Schistura*	*shuangjiangensis*	JF340404
*Paracobitis*	*anguillioides*	HM010582
*Triplophysa*	*xiangxiensis*	JN696407
	*stoliczkai*	DQ105249
	*siluroides*	EF212443
	*bleekeri*	FJ406605
	*stenura*	JN837657
	*orientalis*	DQ105251
*Nemacheilus*	*maysae*	GQ174377
	*ornatus*	GQ174363
	*pallidus*	GQ174370

## Results

### Oreonectes daqikongensis sp. n.

Taxon classificationAnimaliaCypriniformesNemacheilidae

http://zoobank.org/598D9793-208A-45ED-BCA9-A1C9203003A3

#### Type materials.

The 26 specimens were collected from Daqikong area of Libo County, Guizhou; the overall length of the specimen was 37.82–83.10 mm and the body length was 31.28–70.96 mm.

#### Holotype.

**Figure 1.**
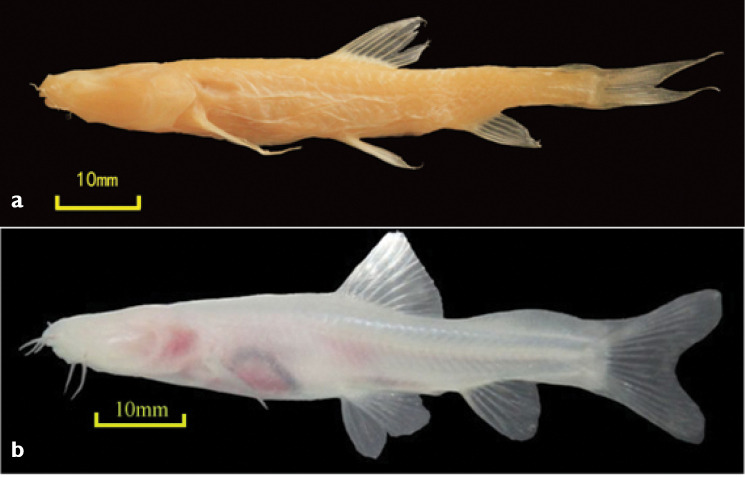
**a** Holotype of *Oreonectes daqikongensis* sp. n. NO.CNGZNU20110128002. **b** A living *Oreonectes daqikongensis* sp. n.

(No. CNGZNU20110128001; Figure 1a–b) The total length is 77.14 mm and the body length is 61.46 mm. Holotype was collected from a subterranean river of the Daqikong area (N 25°17'05.1", E 107°44'54.3"; H 488 m) in January 2011. It was stored in the animal specimen room of the School of Life Sciences, Guizhou Normal University, Guiyang, China.

#### Paratypes.

(25, No. CNGZNU20110128002–CNGZNU20110128026) Paratypes were collected and stored in the same places as the holotype.

#### Habitat.

**Figure 2.**
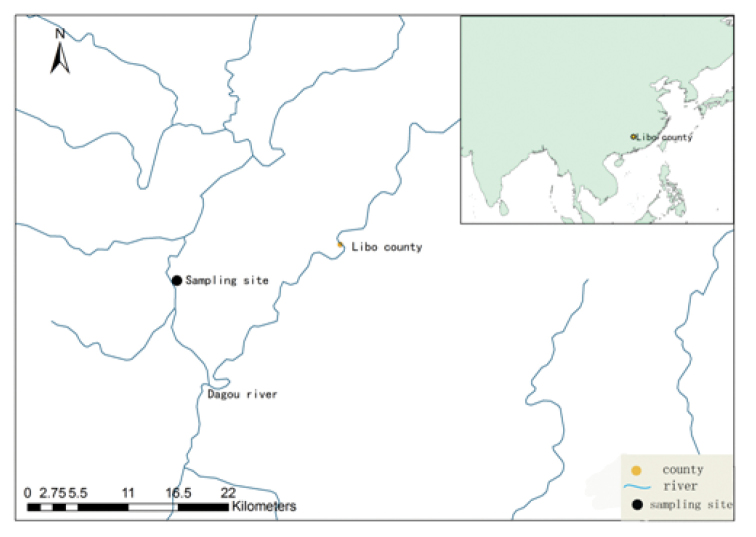
Collection localities of *Oreonectes daqikongensis* sp. n.

This species was found only in the Daqikong scenic area. The opening of the cave was halfway up the mountain, and the distance from the opening to the pool was about 15–20 m. The cave got no sunshine because of the twisty pathway. A large number of *Hipposideros armiger* lived in the cave and a thick layer of bat dung was found on the ground. Groundwater extended into the cave, and the water rushed outside the cave in the case of heavy rain. So far, no other fish, shrimps, or aquatic animals were found in the cave. The subterranean river belonged to the Dagou river system, and was the main river of the Libo County, which runs through the whole county, enters Guangxi from the Laocun Xiang, and was the major tributary of the Duliu River system (Figure 2).

#### Diagnosis.

The species has a large head, and the width of the head is larger than its depth. The frontal torso is nearly cylindrical, the backend gradually compresses, and the head is slightly flattened. There is a short distance between the anterior and posterior nostrils, and the anterior nostril forms a short and tubular structure, which is truncated backward. The pectoral fin extends backward to or beyond the starting point of the pelvic fin. The body is naked. The eyes are completely degraded; and eye socket was filled in fat tissue and without any outside remnant indicating their presence. The superior and inferior caudal peduncles have well-developed soft finfolds. No carneous fin flaps are present in the pelvic fin axilla. The air bladder is wrapped in a bony capsule, and the posterior chamber of the air bladder is developed into a membranous chamber, which is separated from the abdominal cavity and connected to the anterior chamber by a short duct. The whole body is white and transparent, when they are alive, they look a little red because the blood inside, and is unlikely to become black when it is fed in sunlight for a long term.

#### Description.

The species has a slightly elongated body, slightly ridged back, body slightly spindle, and compressed hindquarters, and its widest part is at the gill cover. The main measurable characters of *Oreonectes daqikongensis* are shown in Table 2. The head is slightly flattened, lips are rounded, eyes vestigial. Anterior and posterior nostrils well separated. Anterior nostril forms short tubular and posterior nostrils elliptical. The fish has an inferior mouth, and the upper and lower lips are connected at the corner of the mouth. The upper jaw is curved and the lower jaw is spoon shaped. The mouth aperture is U-shaped, and its rear end reaches the bottom part of the posterior edge of the naris. It has three pairs of slim barbels, and one pair each of inner rostral barbels, outer rostral barbels, and mouth corner barbels. The inner rostral barbels are shorter, and the outer rostral barbels extend backward to exceed the rear edge of the posterior naris, while the maxillary barbels extend backward and their ends are appropriate at the center of the rear edge of the opercular bone. The superior and inferior sides of the caudal peduncle have well-developed ridge-like fatty soft fin folds; especially, the soft fin folds between the superior caudal peduncle and the dorsal fin are more apparent than those in the inferior caudal peduncle, where its front end reaches the upper part of the anal fin. The superior soft fin folds originate from the rear edge of the dorsal fin base to one third of the front edge of the caudal fin base, where compressing the dorsal fin backward can reach the origin of the soft fin folds. The inferior soft fin folds originate from the rear edge of the anal fin base to one third of the front edge of the caudal fin base.

**Table 2.** Main morphometric characters of *Oreonectes daqikongensis* sp. n.

Measurements	Holotype	Range	Mean ± SD
Total length (mm)	50.96	41.62~77.14	57.15 ± 10.36
Standard length (mm)	41.68	34.22~61.46	46.49 ± 8.11
Percentage (%) of SL			
Body height	18.04	15.30~23.26	20.43 ± 1.92
Body width (at dorsal fin origin)	13.72	12.42~19.74	15.86 ± 1.77
Predorsal length	50.82	50.79~56.32	53.06 ± 1.74
Length of caudal peduncle	18.67	13.16~19.18	16.50 ± 1.95
Depth of caudal peduncle	7.15	7.15~9.90	8.26 ± 0.82
Percentage (%) of HL			
Body height	60.74	56.94~82.90	70.48 ± 6.93
Head height (at nape)	44.75	44.30~58.84	50.19 ± 4.38
Head width	49.11	49.11~61.73	55.47 ± 4.03
Length of inner rostral barbel	17.12	12.36~17.75	15.03 ± 1.83
Length of outer rostral barbel	24.39	18.42~33.12	25.86 ± 4.29
Length of maxillary barbel	25.85	22.92~30.17	27.04 ± 1.68
Percentage (%) of TL			
Body height	14.76	12.69~18.77	16.43 ± 1.57
Body width(at dorsal fin origin)	11.22	10.30~15.91	12.90 ± 1.36
Head length	24.29	22.29~25.93	23.62 ± 0.93

The distance from the dorsal fin origin to the rostral end is larger than that from the dorsal fin origin to the caudal fin base, and the outer edge is truncated or slightly concave. The rear end of the dorsal fin can be compressed to reach the soft fin fold origin. The fish has a long pectoral fin, which extends backward to or beyond the pelvic fin base. Also, the pectoral fin has a very special morphology, which does not have branched fin rays. The first and second fin rays are very long, forming a spoke-like shape. The ventral fin originates at a place opposite to the origin of the dorsal fin, and it extends backward to cover the anus and close to the origin of the anal fin. The distance from the anus to the anal fin origin is about 1 mm. The anal fin extends to its base to half of the caudal fin base. The posterior edge of the caudal fin is forked, and the upper lobe is slightly longer than the lower lobe.

The fish is naked, and the intact lateral line is superficially subcutaneously buried, which is flattened from the upper angle of the gill cover and extends backward to the center of the caudal fin base. Sensory tubes are present in the head connecting to the lateral line at the upper part of the posterior edge of the gill cover, and bifurcate into two lateral lines toward the head. These tubes travel from the supraorbital bone to the inner side of the anterior nostrils and from the infraorbital bone to the outer side of the nostrils, and connect to the two lateral lines via a transverse lateral line at the parietal bone. The whole body is colorless, and the living fish is translucent, where the internal organs are visible. The stomach is U-shaped, and the intestine is in its rear part, which is slightly curved and extends to the anus. The anterior bladder chamber is completely coated by the bony bladder sac, which has a bony posterior wall and no opening. However, the posterior bladder is a well-developed membranous chamber, which is separated from the abdominal cavity and connected to the anterior chamber by a short tube. An oval transparent area exists in the posterior branchial aperture, which is inset in both sides of the body.

#### Color.

The whole body of the living species is pale pink and translucent, where the vertebra, body segment at caudal peduncle, cardinal gill, and internal organs are visible. Its body color is unlikely to change when it is fed in the laboratory under light for a long term.

#### Phylogenetic findings.

**Figure 3.**
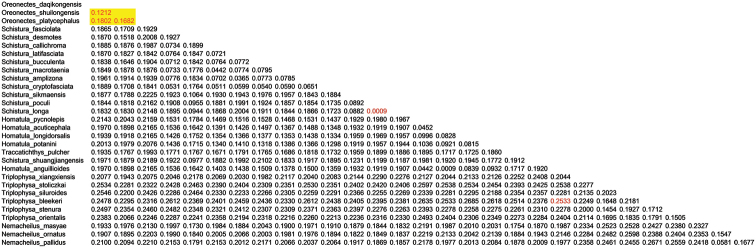
Genetic distance between *Oreonectes daqikongensis* sp. n. and species of Nemacheilinae.

**Figure 4.**
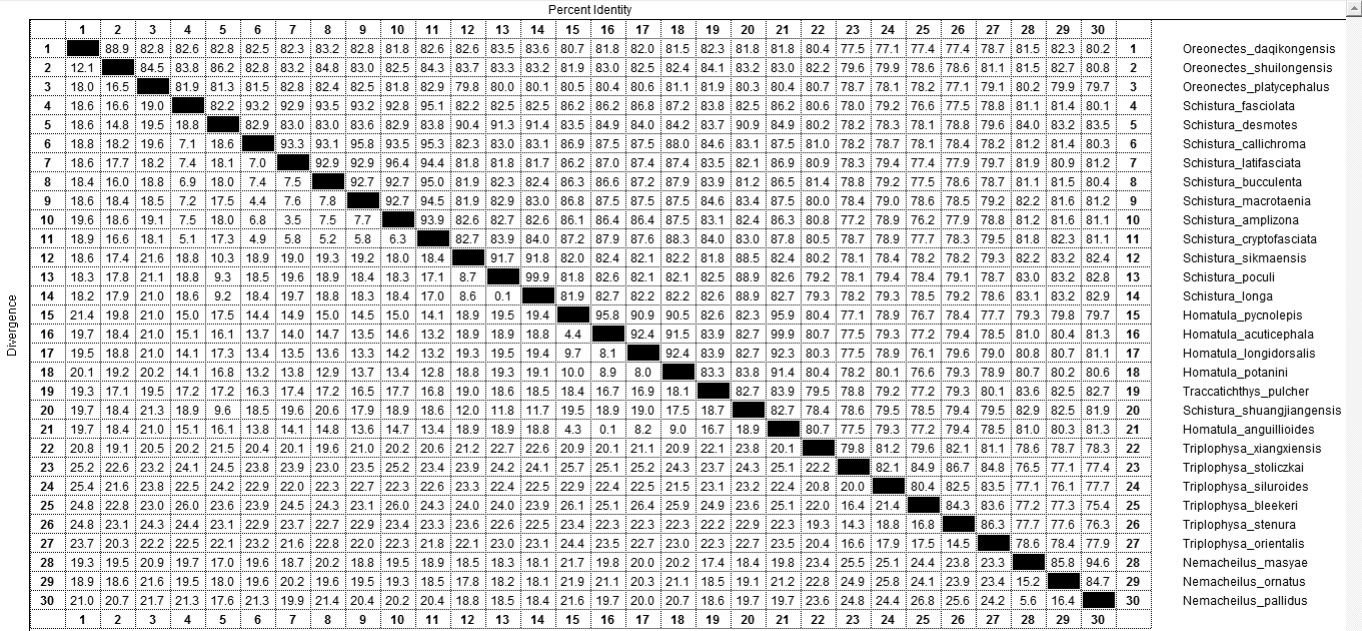
The sequence divergence of Cyt b between *Oreonectes daqikongensis* sp. n. and species of Nemacheilinae.

**Figure 5.**
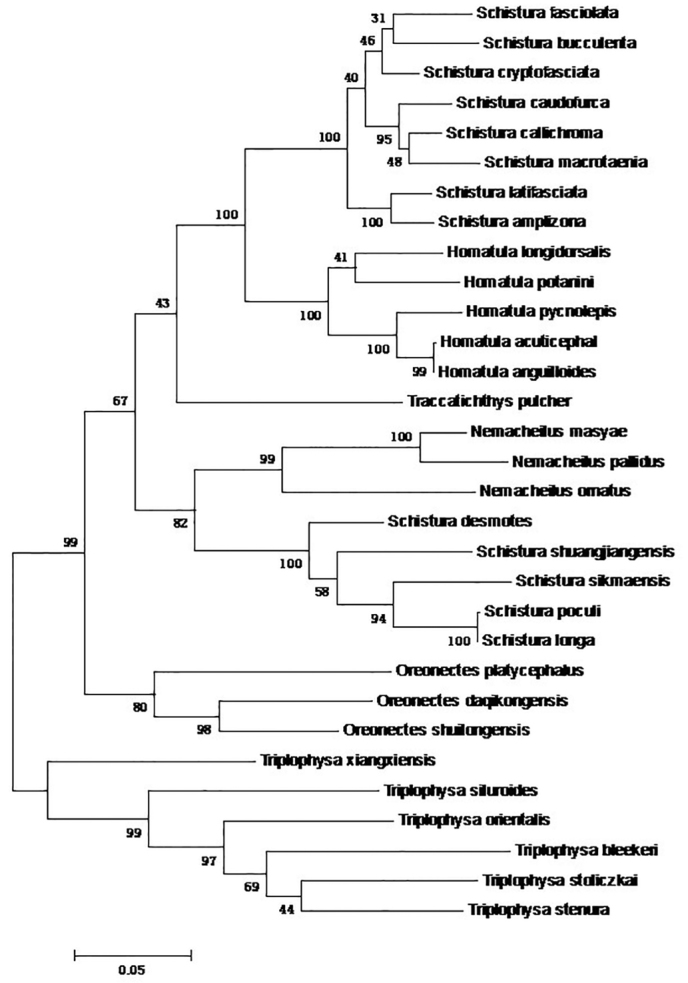
Maximum likelihood phylogenetic trees of Nemacheilinae.

In the 25 specimens of *Oreonectes daqikongensis* sp. n., the cyt b sequence was at 1140 bp, and the base did not show any difference among them, in which *T* = 27.6%, *C* = 28.7%, *A* = 28.8%, and *G* = 14.9%, and the overall transition/transversion rate was *R* = 0.50. The genetic distances between the new species and *Oreonectes platycephalus* and *Oreonectes shuilongensis* were 0.1802 and 0.1212, respectively, which were smaller than the genetic distance among species of other categories. The genetic distance between *Oreonectes* and the other categories of Nemacheilinae ranged from 0.1518 to 0.2546. The interspecific genetic distance of Nemacheilinae was 0.0009–0.2533(Figure 3). The sequence divergence of Cyt b between this species and *Oreonectes shuilongensis* was 13.7, and that between this species and *Oreonectes platycephalus* was 19.4. Additionally, the divergence ranged from 19.8 to 27 between this species and species of other genus of the Nemacheilinae subfamily (Figure 4). The divergence of *Oreonectes* was smaller compared with the other genus of Nemacheilinae. Since the genetic distances between *Oreonectes daqikongensis* sp. n. and the other species of *Oreonectes* were greater than the interspecific distance of each category of Nemacheilinae, *Oreonectes daqikongensis* sp. n. was considered as a new species. In the phylogenetic tree (Figure 5), *Oreonectes daqikongensis sp. n* was clustered with *Oreonectes shuilongensis* (Bootstrap value (BP) = 99) and *Oreonectes platycephalus* (BP = 74). *Oreonectes* with *Schistura*, *Homatula*, and *Nemacheilus* genera was divided into two subsets (BP = 97). This species inhabits in the karst caves. Cave environments were easier to form geographical isolation for the independence of different caves. Therefore, according to the genetic distance, differences in sequences, and phylogenetic tree analysis, *Oreonectes daqikongensis* sp. n. belongs to a new species of *Oreonectes*.

## Discussion

This species has nearly cylindrical forequarters, gradually compressed hindquarters, and a slightly flat head. The anterior and posterior nostrils are separated by a short distance, and the anterior nostrils form a short and tubular structure with their rear ends extending to become whisker or cusp. It has three pairs of barbels, and naked body. The anterior bladder chamber is completely coated by the bony bladder sac, and the posterior bladder is a well-developed membranous chamber, which is separated from the abdominal cavity and connected to the anterior chamber by a slender duct. Its stomach is U-shaped. All these features are consistent with the typical characteristics of *Oreonectes* described by Zhu (1989).

The new species has completely degraded eyes, without any outside remnant indicating their presence. This feature differed from the features of the following four species: *Oreonectes donglanensis* and *Traccatichthys xiangxiensis* had degraded eyes, with only a small black spot visible, and the eye sockets are filled with loose fat globules; *Oreonectes macrolepis* had very small eyes, almost blind; and *Oreonectes microphthalmus* had highly degraded eyes, with only eyespots or eye sockets visible. This feature can also be differentiated from the features of the following five species that have normal eyes in appearance: *Oreonectes platycephalus*, *Oreonectes polystigmus*, *Oreonectes luochengensis*, *Oreonectes retrodorsalis*, and *Oreonectes elongates*.

Forked caudal fins of the new species, so the new species belong to furcocaudalis type. Eyes completely degradation of this new species can be different from other species of furcocaudalis group with eyes nomal. Such as *Oreonectes microphthalmus*, *Oreonectes macrolepis*, *Oreonectes retrodorsalis*, *Oreonectes duanensis*, *Oreonectes donglanensis* and *Oreonectes furcocaudalis*. Lateral line complete of the new species, this characteristic make a distinction between *Oreonectes barbatus*, *Oreonectes elongatus*, *Oreonectes translucens* and *Oreonectes acridorsalis* which are lateral line incomplete or no lateral line. The comparison of main traits between the new species and the similarity species of *Oreonectes* (*Oreonectes barbatus*, *Oreonectes elongatus*, *Oreonectes translucens* and *Oreonectes acridorsalis*) is in Table 3.

**Table 3.** Comparison of traits between *Oreonectes daqikongensis* sp. n. and the similarity species of *Oreonectes*.

Trait	*Oreonectes daqikongensis* sp. n.	*Oreonectes acridorsalis*	*Oreonectes barbatus*	*Oreonectes elongatus*	*Oreonectes translucens*
No.	15	5	8	3	3
Locality of collection	Daqikong area, Libo County, Guizhou	Tian’e County, Guangxi	Nandan County, Guangxi	Huangjiang County, Guangxi	Duan County Guangxi
Dorsal fin rays	iii, 8–9	iii, 7	iii, 8–10	iii, 8–9	iii, 8
Anal fin rays	iii, 6	iii, 5	ii, 5–6	ii, 6–7	iii, 6
Pectoral fin rays	i, 11–12	i, 10–12	i, 10–12	i, 10	i, 11
Pelvic fin rays	i, 6–7	i, 5–6	i, 6	i, 6	i, 6
Caudal fin branched rays	13–14	13–14	14	14	16
Pectoral fin rays	Pectoral fin extend to or beyond the origin of the ventral fins	Pectoral fin Fan-shaped, extend to half of the distance between origins of pectoral fin and ventral fin	Pectoral fin extend to approx. two thirds of the distance between origins of pectoral fin and ventral fin	Pectoral fin long and narrow, greater than 1/2 the distance between origins of pectoral and pelvic fins	Pectoral fin long and narrow, almost reaching pelvic-fin origin
Pelvic fin rays	The first and second branched rays of ventral fin are long, but do not form a spiny shape	Pelvic fin is shorter, origin is ahead of dorsal fin origin, extend, but cannot reach anus	Pelvic fin Origin is opposite to the dorsal fin origin, extend but cannot reach the anus	Pelvic fin relatively slender, extending slightly over anus	Pelvic fin extending slightly beyond anus.
Anal fin rays	Anal fin extend to reach half between the anal fin base and caudal fin base	Anal fin truncated outer edge	Anal fin truncated or slightly convex outer edge	Anal fin origin next to anus, tip nearly reaching middle of caudal peduncle	Anal-fin origin just posterior to anus
Lateral line	Lateral line is subcutaneously buried completely, which in the head has three branches	No lateral line in the body, lateral line in the head is not well-developed	No lateral line in the body, lateral line in the head is not well-developed	Lateral line incomplete, with 4 pores behind opercle, connecting to the cephalic lateral-line system.	Lateral line incomplete, with only 3 or 4 lateral pores behind head.
Eyes	Absent	Absent	Absent	Absent	Absent

*Oreonectes daqikongensis* sp. n. and *Oreonectes shuilongensis* are both distributed in Guizhou province, but in different county. The two species which have close genetic distance. There are different characteristics as following: a forked caudal fin (vs. truncated or slightly concave belong to platycephalus group), possessing adipose crests of the caudal peduncle (vs. no adipose crests), disappeared eye (vs. eye normal), lateral line is completely (vs. incomplete, 8–10 pores) and body translucence (vs. top of head and body gray and black). *Oreonectes daqikongensis* sp. n. and *Oreonectes platycephalus* which have close genetic distance with the new species. However, they can be differs by naked body (vs. scaled body), disappeared eye (vs. eye normal) and anterior nostril in a short tubular structure, which is obliquely cut tube (vs. short tube extending into relatively long barbel, beyond edge of posterior nostril) (Table 4).

**Table 4.** Comparison of traits between *Oreonectes daqikongensis* sp. n. and similar species of *Oreonectes*.

Trait	*Oreonectes daqikongensis*	*Oreonectes shuilongensis*	*Oreonectes platycephalus*
No. of specimens	15	16	4
Location	Libo, Guizhou	Sandu, Guizhou	Zhaoping, Guangxi
Dorsal fin rays	iii, 8–9	iii, 7–8	iii, 6–7
Anal fin rays	iii, 6	iii, 6	iii, 5
Pectoral fin rays	i, 11–12	i, 11–12	i, 10
Pelvic fin rays	i, 6–7	i, 6	i, 6–7
Anterior nostril	Anterior nostril in short tubular structure, which is obliquely cut tube	Anterior nostril in short tube extending into relatively long barbel, beyond posterior edge of eye	Anterior nostril in short tube extending into relatively long barbel, beyond edge of posterior nostril
Lateral line	Lateral line is subcutaneously buried completely, which in the head has three branches	Lateral line incomplete, terminates above pectoral fin; 8–10 pores	Lateral line incomplete, terminates above pectoral fin
Caudal fin	caudal fin is forked	Truncated or slightly concave	Rounded
Body color	The whole body of the living species is pale pink and translucent	Top of head and body gray and black in fresh condition; grayish in dorsum and body light brown after preservation in alcohol; fins transparent	In formaldehyde, body light brown; dorsum and side of body with irregular dark gray; dark brown horizontal stripe at end of caudal fin; fins without stains

Both morphological and molecular phylogenetic evidence revealed that *Oreonectes daqikongensis* sp. n. is a new species of *Oreonectes*. *Oreonectes* is distributed in the underground rivers in the karst region of Southwest China, of which *Oreonectes platycephalus* is most widely distributed. All these areas belong to the Pearl River and the Red River systems. Most of this species is distributed in the Karst regions of Guangxi. Currently, *Oreonectes shuilongensisis* and *Oreonectes daqikongensis* sp. n. have been discovered in Guizhou, and most are distributed in the southern area of Guizhou. This work is the first time to descript the new species in detail. The discovery of this species will be conducive to comprehensively understand the distribution of *Oreonectes*.

## Supplementary Material

XML Treatment for Oreonectes daqikongensis

## Appendix 1

**Table:** The source of the material of other *Oreonectes* species.

Species	Number	Standard length (mm)	Distributions locations	River system
*Oreonectes platycephalus*	KIZ200304597–200304600	50.9~58.6	Jinxiu county, Guangxi	Zhujiang
*Oreonectes polystigmus*	KIZ2001060507 (holotype)	57.7	Guilin city, Guangxi	Guijiang
*Oreonectes microphthalmus*	KIZ2004009395 (holotype)	39.0	Du’an county, Guangxi	Red River
*Oreonectes macrolepis*	CLJH91065 (holotype)	54.8	Huanjiang county, Guangxi	Liujiang
*Oreonectes retrodorsalis*	9110001 (holotype)	37.00	Nandan county, Guangxi	Red River
*Oreonectes acridorsalis*	CLJH04100608 (holotype)	48.3	Tian’e county, Guangxi	Red River
*Oreonectes barbatus*	CLJH2009080003 (holotype)	54.1	Nandan county, Guangxi	Liujiang
*Oreonectes duanensis*	CLJH2011090302 (holotype)	56.1	Du’an county, Guangxi	Red River
*Oreonectes donglanensis*	CLJH2010010051(holotype)	45.2	Donglan county, Guangxi	Red River
*Oreonectes elongatus*	ASIZB189288 (holotype)	78.2	Huanjiang county, Guangxi	Zhujiang
*Oreonectes guananensis*	KIZ2010003067 (holotype)	77.0	Huanjiang county, Guangxi	Xijiang
*Oreonectes luochengensis*	KIZ2010003073 (holotype)	74.9	Luocheng county, Guangxi	Xijiang
*Oreonectes translucens*	ASIZB94785 (holotype)	45.8	Du’an county, Guangxi	Red River
*Oreonectes furcocaudalis*	KIZ9309003, KIZ9309004	58.0~58.7	Du’an county, Guangxi	Red River
*Oreonectes anophthalmus*	KIZ1994001-005	25.3~36.9	Wuming county, Guangxi	Youjiang
